# Supplementary material for: Development of Spirulina-Enriched Fruit and Vegetable Juices: Nutritional Enhancement, Antioxidant Potential, and Sensory Challenges
Source: Foods. 2025 Oct 17;14(20):3539. doi: 10.3390/foods14203539 (PMC12563328; doi:10.3390/foods14203539)
Supplement: Supplementary file 1 [file foods-14-03539-s001.zip › foods-3937644-supplementary.pdf]

Supplementary Table S1: The results of initial acceptability test (seven-point hedonic scale) of juices; juices marked in bold were used in further study

Sample	Score	Sample	Score
Apple	5.40	**Tomato**	**6.00**
Apple + 2 g blue	3.20	**Tomato + 2 g blue**	**5.00**
Apple + 2 g green	2.60	Tomato + 2 g green	3.20
Apple + 4 g blue	2.20	**Tomato + 4 g blue**	**4.00**
Apple + 4 g green	3.00	Tomato + 4 g green	3.60
**Sour cherry**	**6.50**	Celery	4.00
**Sour cherry + 2 g blue**	**5.25**	Celery + 2 g blue	1.00
Sour cherry + 2 g green	4.25	Celery + 2 g green	1.00
**Sour cherry + 4 g blue**	**5.00**	Celery + 4 g blue	1.00
Sour cherry + 4 g green	3.75	Celery + 4 g green	1.00

Supplementary Table S2: Sensory descriptors, their definitions and assessment techniques

Property	Descriptor	Technique	Definition
Appearance	Blue colour intensity	Visually	Intensity of juice blue colour
	Turbidity	Visually	Turbidity of juice (0 – clear juice; 100 – cloudy juice)
Texture	Viscosity	Visually	Juice viscosity (0 – water; 100 – tomato puree)
	Granularity	Visually	Presence of visible particles in juice (0 – clear water; 100 – bran dispersed in water)
Odour	Characteristic odour	Olfactory	Intensity of odour characteristic for sour cherry/tomato juice
	Algae odour	Olfactory	Intensity of odour characteristic for microalgae
Flavour	Characteristic flavour	Orally	Intensity of flavour characteristic for sour cherry/tomato juice
	Algae flavour	Orally	Intensity of flavour characteristic for microalgae
Taste	Sweet	Orally	Basic taste produced by sugars such as sucrose, fructose, glucose (0 – water; 100 – syrup)
	Salty	Orally	Basic taste produced by sodium salts, such as sodium chloride, and in part by other salts, such as potassium chloride (0 – water; 100 – 4% NaCl solution)
	Sour	Orally	Basic taste produced by acid substances such as citric, acetic and lactic acid (0 – water; 100 – orange juice)
	Bitter	Orally	Basic taste produced by substances such as caffeine and quinine (0 – water; 100 – black coffee)
